# Role of Oxidative RNA Damage in Chronic-Degenerative Diseases

**DOI:** 10.1155/2015/358713

**Published:** 2015-05-20

**Authors:** Carmela Fimognari

**Affiliations:** Department for Life Quality Studies, Alma Mater Studiorum-University of Bologna, 47921 Rimini, Italy

## Abstract

Normal cellular metabolism and exposure to ionizing and ultraviolet radiations and exogenous agents produce reactive oxygen species (ROS). Due to their reactivity, they can interact with many critical biomolecules and induce cell damage. The reaction of ROS with free nucleobases, nucleosides, nucleotides, or oligonucleotides can generate numerous distinct modifications in nucleic acids. Oxidative damage to DNA has been widely investigated and is strongly implicated in the development of many chronic-degenerative diseases. In contrast, RNA damage is a poorly examined field in biomedical research. In this review, I discuss the importance of RNA as a target of oxidative damage and the role of oxidative damage to RNA in the pathogenesis of some chronic-degenerative diseases, such as neurological disorders, atherosclerosis, and cancer. Furthermore, I review recent evidence suggesting that RNA may be the target for toxic agents and indicating RNA degradation as a powerful tool to treat any pathology in which there is an aberrant expression of mRNA and/or its gene products.

## 1. Introduction

Under most conditions, normal cellular metabolism is the source of endogenous reactive oxygen species (ROS). Multiple steps in the electron transport chains involve the formation of the O_2_^•−^. Some enzyme activities generate O_2_^•−^ and ROS are released from phagocytes. ROS can also be produced by ionizing and ultraviolet radiations and exogenous agents following a redox cycle induced by cell metabolism. ROS play a physiological role as they are involved in destroying cells infected with viruses or bacteria [1] and in cell signaling processes [2]. However, due to their reactivity, they can interact with many critical biomolecules and induce cell damage [3]. Oxidative damage to DNA has been widely investigated and is strongly implicated in the development of many chronic-degenerative diseases [4, 5]. In contrast, RNA damage is a poorly examined field in biomedical research.

In this review, I discuss the involvement of RNA in the oxidative damage and the critical role that oxidative damage to RNA plays in the pathogenesis of several chronic-degenerative diseases. Furthermore, I review recent studies reporting that RNA may be the target for different toxic agents and identifying RNA inactivation as an effective strategy to treat any pathology where an aberrant expression of mRNA and/or its gene products is observed.

## 2. Oxidative Damage to RNA

Under most conditions, generation of ROS is due to metabolic reactions and is a part of the aerobic energy building process. The main source of ROS in human cells is mitochondria, where the rate of ROS production is proportional to the rate of mitochondrial respiration [6].

At low levels, ROS play a pivotal role in cell division and survival, cell signaling, inflammation and immune functions, autophagy, and stress response. However, a redox imbalance caused by exposure to oxidant agents alters a biological system's ability to detoxify ROS or to repair any damage caused by them [6].

Damage to RNA can be produced by HO^•^ rising from O_2_^•−^ and H_2_O_2_ by the Fenton and Haber-Weiss reactions. The reaction of ROS with free nucleobases, nucleosides, nucleotides, or oligonucleotides can generate numerous distinct modifications in nucleic acids. Many different types of oxidatively altered bases have been recorded in DNA [3, 7]. Guanine has the lowest standard reduction potential. Thus, it represents the major site of oxidative damage in DNA [8]. The most frequent detected lesions are 8-hydroxyguanine, 8-hydroxyadenine, 2,6-diamino-4-hydroxy-5-formamidoguanine, 4,6-diamino-5-formamidoadenine, and cytosine glycol [9].

Although both HO^•^ and H_2_O_2_ can damage RNA [10], 8-hydroxyguanosine is the only oxidized base characterized so far in RNA [8], thus indicating that this common oxidative modification to DNA occurs in RNA as well. The formation of 8-hydroxyguanosine occurs by reaction of guanine with HO^•^ followed by oxidation or by reaction of guanine with singlet oxygen followed by reduction [11]. This oxidative modification is also the most deleterious since 8-hydroxyguanosine can potentially alter genetic information by incorrectly pairing with adenine at similar or higher efficiency than with cytosine in RNA and produce mutations at the level of transcription [12]. Alterations of ribose, base excision, and strand break can represent other modifications induced by ROS on RNA [13].

Oxidative damage can alter RNA structure and function and interfere with the interaction between RNA and other cellular molecules. As an example, oxidative damage to RNA template produces the block of reverse transcription [13]. Moreover, oxidation of mRNA leads to reduced translation efficiency and abnormal protein production [14] and causes ribosome dysfunction [15].

Noncoding RNAs, such as rRNAs and tRNAs, and small nuclear RNAs, have a normal half-life much greater than 3 h. Two different studies reported the presence of 8-hydroxyguanosine in RNA, with a half-life of ~12.5 h [16, 17]. High levels of 8-hydroxyguanosine were observed in neurons within the hippocampus, subiculum, entorhinal cortex, and frontal, temporal, and occipital neocortex in autoptic brain tissues of patients affected by Alzheimer's disease [18]. Similar results were found in old rats with memory loss [19]. Taken together, those observations imply a partial accumulation of damaged RNA in neurodegeneration and aging.

## 3. Underlying Causes of RNA Susceptibility to Oxidative Damage

Cellular RNA could theoretically be more prone to oxidative damage than DNA for different reasons. For instance, mammalian cells have RNA levels 4.4 times higher than that of DNA by weight. RNA is less associated with proteins. Moreover, its bases are not protected by hydrogen bonding and are thus more easily accessible to ROS. RNA has an extensive cytoplasmic distribution in close proximity of mitochondria [18], where the majority of ROS is generated. Levels of oxidized nucleosides have been actually found to be higher in mitochondrial DNA than in nuclear DNA [19].

Consistent with the above reported observations, several studies have shown that the levels of oxidative damage in RNA can be 10–20 times higher than that of DNA of the same source in different models, including rat liver [20, 21], human leukocytes [19], and lung epithelial cells [16]. Moreover, a study quantified nucleic acid damage on livers of rats treated with doxorubicin, an oxidant generator. Doxorubicin treatment resulted in a significant increase in liver RNA oxidation, but no significantly increased DNA oxidation [21]. Similar results were obtained in human lung epithelial cells, where quantification of 8-oxo-7,8-dihydroguanosine content in RNA* versus* 8-oxo-7,8-dihydro-2′-deoxyguanosine content in DNA has been performed following treatment with H_2_O_2_. In this experimental system, the level of RNA damage* per* nucleoside resulted in 14–25 times greater than that in DNA [16].

Another factor that may account for the high susceptibility of RNA to oxidative damage is the iron-binding properties of certain classes of RNAs. Iron catalyzes Haber-Weiss and Fenton reactions that produce ROS [11]. Purified ribosomes from Alzheimer's patients were found to have elevated levels of associated redox-active iron. Moreover,* in vitro* studies demonstrated that rRNA had higher iron binding than tRNA or mRNA. Accordingly, iron-rich rRNA had a 13-fold greater formation of 8-oxo-7,8-dihydroguanosine in oxidation experiments as compared to the iron-poor tRNA [22].

The higher levels of oxidization observed for RNA could also be due to different rates of removal of RNA and DNA damage. Indeed, in contrast to the redundant repair mechanisms for DNA, little is still known about how and to what extent oxidatively damaged RNA is removed [23].

## 4. Mechanisms of Oxidized RNA Clearing

Some observations support the hypothesis that oxidized RNA is not tolerated by cells and can be cleared from the functional RNA pool by RNA surveillance mechanisms. In mammals, the levels of RNA oxidative damage have been found to be reduced in 24–72 h after removal of the oxidative stress insult [19, 24].

Different studies reported mechanisms for the turnover of oxidized free nucleotides and the turnover of damaged RNAs. The polynucleotide phosphorylase, a 3′-5′ exoribonuclease, binds an oligoribonucleotide containing 8-oxo-7,8-dihydroguanosine with a much higher affinity than it binds to an undamaged RNA [25]. Of note, the polynucleotide phosphorylase is mainly localized in mitochondria, where oxidative damage is high, thus further supporting its role in controlling the level of oxidatively damaged RNA [26].

In humans, MTH1, a nucleotide pool sanitization enzyme, hydrolyzes 8-oxoGTP. The resulting 8-oxoGMP cannot reenter the nucleoside triphosphate pool because guanylate kinase lacks activity on 8-oxoGMP [27]. A second human protein of the hydrolase superfamily, Nudix (nucleoside diphosphate linked moiety X), catalyzes the hydrolysis of 8-oxoGTP to 8-oxoGDP, which can be further hydrolyzed by MTH1 [28], thus ensuring transcriptional fidelity despite oxidative damage to nucleotides. The role of the above reported enzymes in protecting RNA pool from oxidative stress is demonstrated by the evidence that MTH1 expression is upregulated in kainate-induced oxidative stress of rat hippocampus. Accordingly, in the same experimental model, MTH1-null mutant rats show significantly higher levels of 8-hydroxyguanosine in hippocampus RNA [24].

Selective degradation of oxidized RNA requires the ability to distinguish between oxidized RNA and normal RNA. RNases lack such selective degradation. Different studies have reported the existence of RNA-binding proteins that specifically recognize oxidatively damaged RNA molecules and effectively target the damaged RNA to degradation by RNases [25, 29, 30]. The human YB-1 protein is a multifunction protein working as an RNA chaperone to target damaged RNA to sequestration and/or degradation. YB-1 is able to bind 8-hydroxyguanosine-containing RNA oligonucleotide [31], thus aiding in the unwinding and winding of RNA duplexes and conferring high resistance to oxidative stress [32].

Mechanisms for the degradation of mRNA have been reported, where ribosome plays a role in recognizing oxidatively damaged mRNA during translation [33]. The human S3 protein binds DNA containing 8-hydroxydeoxyguanosine or abasic residues and cleaves abasic DNA [34]. It has been suggested that S3 may scan mRNA in a similar manner and inactivate or sequester oxidatively damaged mRNAs [29].

Stable RNAs represent the majority of cellular RNA and thus should contain the majority of damaged lesions in RNA pool. For rRNA, some studies report degradation following oxidative damage. As an example, Martinet et al. have shown that a reduction of 18S and 28S rRNA levels parallels high 8-hydroxyguanosine RNA levels in human atherosclerotic plaques [35]. Similarly, total RNA and ribosomes from patient brains affected by Alzheimer's disease shown decreased levels of rRNAs [36]. However, it is still unclear whether the observed rRNA degradation was an effect of ongoing apoptosis or a specific mechanism to degrade damaged RNAs.

For tRNA, studies on yeast and human cells have found that oxidative stress induces the accumulation of cleaved tRNA halves [37]. However, since mature tRNA levels are not decreased, it is not clear whether these cleavages affect cell function.

## 5. Oxidative RNA Damage in Chronic-Degenerative Diseases

**Figure 1:**
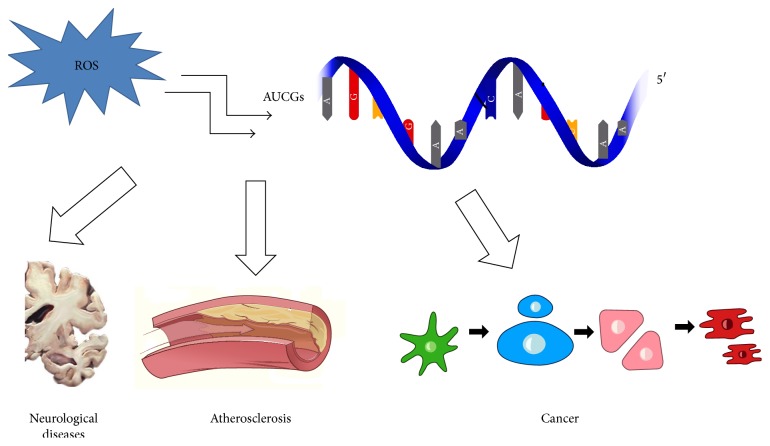
Oxidative RNA damage plays a role in different chronic-degenerative diseases.

Oxidative RNA damage has been recently found to be involved in the pathogenesis of several human diseases, especially chronic degeneration (Figure 1). Oxidative RNA damage has been observed in neurodegenerative diseases including Alzheimer's disease, Parkinson's disease, dementia with Lewy bodies, and prion diseases [38]. Recently, Nunomura et al. analyzed neuronal RNA oxidation in the cerebral cortex of individuals in the transitional stages from normal elderly to the onset of Alzheimer's disease. A slight level of RNA oxidation was observed during the process of aging; a marked level of RNA oxidation was recorded in the transition from normal aging to Alzheimer's disease [39].

An increased level of oxidative DNA and RNA damage and alteration of ribosome function was reported in postmortem brains of patients with Alzheimer's disease and Parkinson's disease [15, 18, 40]. By using specific antibodies, 8-hydroxydeoxyguanosine and 8-hydroxyguanosine were detected mainly in the cytoplasm of vulnerable neurons. This observation indicates either mitochondrial DNA or cytoplasmic RNA as major targets of oxidative damage. Treatment with a mixture of both DNases and RNase abolished 8-hydroxydeoxyguanosine and 8-hydroxyguanosine immunoreactivity. However, the immunoreactivity was greatly reduced after RNase, whereas DNase I treatment did not modify either the intensity or the distribution of the immunoreactivity. On these bases, it was concluded that oxidized nucleoside was predominantly associated with RNA rather than DNA [18]. Furthermore, microscopic observation showed that most of 8-hydroxyguanosine was localized to ribosomes [41, 42]. Of note, RNA oxidative damage was greatest early in the disease and decreased with disease progression, thus suggesting that increased oxidative damage is an early event in Alzheimer's disease. On the basis of those findings, Alzheimer's disease seems to be associated with compensatory changes that contrast damage from reactive oxygen [41].

Notably, the presence of 8-hydroxyguanosine was also observed in cerebrospinal fluid from patients with Alzheimer's disease and Parkinson's disease [43] and in serum of Parkinson's disease patients [44].

On the whole, the observations above reported could suggest the use of 8-hydroxyguanosine as a biomarker to catch some neurodegenerative disorders, such as Alzheimer's disease, in their early phase.

Similar RNA oxidative damage was observed in brain samples of patients with dementia with Lewy bodies [45], Creutzfeldt-Jakob disease [46], and subacute sclerosing panencephalitis [47].

Of particular interest, different studies detected a regional distribution of RNA oxidative damage that mirrors the selective neuronal vulnerability that characterizes each neurological disease. Indeed, an increase in the levels of 8-hydroxyguanosine was observed in the hippocampus and cerebral neocortex in Alzheimer's disease, in the* substantia nigra* in Parkinson's disease, and in the motor cortex and spinal cord in amyotrophic lateral sclerosis; no alteration in 8-hydroxyguanosine levels was detected in the cerebellum in Alzheimer's disease, Parkinson's disease, and amyotrophic lateral sclerosis compared with the controls [15, 18, 40, 48–51]. Further studies reported that oxidative RNA damage was predominantly localized in neurons compared with glial cells [51].

The analysis of* postmortem* tissues from patients with Alzheimer's disease and amyotrophic lateral sclerosis evidenced that mRNA oxidation does not represent a random phenomenon but is highly selective. The hypothesis that the selective mRNA oxidation was due to the abundance of mRNA species was excluded. Indeed, although *β*-actin and MAP-2 mRNAs are abundant mRNA species, only very small amounts of their oxidized products were detected [4].

Oxidative RNA damage can cause an impairment of translation due to both mRNA damage and decreased ribosome function. The observed correlation between oxidation of some mRNAs in affected neurons of patients affected by Alzheimer's disease and low protein expression for those genes [52] lends further support to this latter notion. Moreover, oxidatively damaged mRNAs are translated in proteins forming aggregates or generate truncated proteins, which must be degraded by the proteasome [53].

Ribosome stalling is another consequence of mRNA oxidation [52], as suggested by the decreased rate of protein synthesis reported for polyribosomes isolated from neurons vulnerable to oxidative damage of patients with Alzheimer's disease compared to polyribosomes from healthy neurons [15].

Epilepsy is a chronic condition whose incidence increases with age [54]. Recently, it has been reported that epilepsy represents a clinical picture of Alzheimer's disease [55]. A study has recently demonstrated the involvement of RNA oxidation in epileptogenesis. Shortly after the induction of epilepsy by pilocarpine, a significant increase in RNA oxidation was detected in vulnerable neurons. Accordingly, daily supplementation of coenzyme Q10 decreased RNA oxidative damage and protected against pilocarpine-induced seizure activity [4].

The involvement of RNA mutations induced by ROS has been suggested as a possible initial event in the etiology of sporadic prion disease. The hypothesis is based on the evidence that, in Prp-expressing cells, mutations occur in the Prp mRNA more than in the corresponding two copies of the Prp gene. Moreover, the lack of RNA repairs systems similar to those found for DNA mismatch correction results in a higher rate of RNA mutations. Thus, RNA mutations induced by ROS could trigger miscoding that can transiently generate Prp^Sc^ and lead to the propagation of the disease-associated conformational changes [56]. In this regard, it is important to emphasize that oxidative stress enhances Prp^C^ protein expression* in vitro* [57] and would favor the synthesis of unglycosylated Prp isoforms [58] and thus the propagation of the conformational change.

In addition to neurological diseases, oxidative RNA damage has been described in advanced human atherosclerotic plaques [35, 59]. In atherosclerotic artery samples from carotid endarterectomies, 8-hydroxyguanosine was found in the entire plaque in smooth muscle cells, macrophages, and endothelial cells, but not in smooth muscle cells of adjacent normal media or in mammary arteries. This observation was further supported by the decrease in 8-hydroxyguanosine immunostaining after pretreatment with RNase but not after treatment with DNase. The presence of oxidative RNA damage could interfere with protein synthesis. Indeed, mRNA damage could cause abnormal protein translation, and tRNA and rRNA damage could cause dysfunction of protein synthesis. Taken together, those observations suggest that RNA damage could lead to destabilization and rupture of atherosclerotic plaques [59].

Although, preliminary, some experimental observations suggest a potential involvement of oxidative RNA damage in cancer development [60]. 2-Nitropropane is a carcinogenic compound [61] with different industrial applications [62]. Oxidative denitrification of 2-nitropropane is catalyzed by cytochrome P450 through the formation of a 2-hydroxy-2-nitropropane intermediate [60]. In male rats, the administration of 2-nitropropane induced a 3.6-fold and 11-fold increase in oxidative DNA and RNA damage, respectively [20]. Female rats were less susceptible to the carcinogenic effect of 2-nitropropane [60]. Consistent with this observation, less RNA and DNA modifications were detected in female rats than in male rats. Furthermore, RNA and DNA modifications were observed in liver, which represents the target tissue for 2-nitropropane [60], whereas they were very low in nontarget organs [63] and were minimal in rabbits [64], characterized by a refractory nature to 2-nitropropane carcinogenic effects [65].

Excessive exposure to solar UVB radiations is a key risk factor for skin cancer in humans, operating through DNA damage (e.g., dimers of thymine), damage repair, and photoimmunosuppression [66]. A very recent study found that UVB exposure induces the release of RNA from keratinocytes, which in turn stimulates TNF-*α* and IL-6 production from nonirradiated keratinocytes and peripheral blood mononuclear cells. The release of cytokines from nonirradiated cells was due to UVB-induced modifications in the double-stranded domains of some noncoding RNAs of irradiated keratinocytes and was dependent on Toll-like receptor 3 (TLR3) and Toll-like receptor adaptor molecule 1 (TRIF) [67]. Taking into account the pivotal role that TNF-*α* plays in apoptosis, inflammation, and immune surveillance [68], the TLR3-mediated cytokine release could have important implications for tumorigenesis. Thus, UVB-induced damage to noncoding RNA could act as a damage-associated molecular pattern. Moreover, some observations have been made that are consistent with the formation of RNA photoproducts and thus with the role of UVB-induced RNA damage in skin tumorigenesis. Exposure to physiologically relevant UVB irradiation produces RNA-RNA cross-linking and formation of pyrimidine dimers and 8-hydroxyguanosine [11].

## 6. Closing Remarks

A growing collection of findings suggests that high levels of RNA oxidative damage may significantly affect its function and cause detrimental effects to cells and organisms. The existence of mechanisms to control the levels of RNA oxidation detected from bacteria to humans indicates that RNA damage is a significant issue. However, the molecular details about RNA oxidative damage and its consequences remain to be explored as well as the* in vivo* significance of the mechanism of RNA repair. In some cases, the functional consequence of RNA damage has been explored and includes reduced protein synthesis as a result of ribosomal cross-linking and/or mRNA oxidation. As RNA damage has been found to be associated with different chronic-degenerative diseases, a better understanding of how RNA damage impacts cell function is needed. Recently, different classes of small noncoding RNAs such as miRNAs, siRNAs, and piRNAs have been discovered. They are not translated into proteins but act through complementary base pairing with target RNAs [69]. Along this line, it would be important to explore the functional consequences of RNA damage on small noncoding RNAs.

From a toxicological point of view, the emerging role that oxidative RNA damage can play in several chronic-degenerative diseases should be carefully considered and encourage the inclusion of the analysis of RNA damage in current testing strategies for better defining the toxicity profile of xenobiotics. Interestingly, some recent studies showed that cellular RNA is a sensitive target for different xenobiotics with different mechanisms of action, such as H_2_O_2_, an oxidizing agent that also raises the levels of PtdIns(3,4,5)P_3_ and activates downstream signaling; doxorubicin, which acts as both an alkylating and an oxidizing agent; spermine, one of the few NO-donors releasing authentic NO and ROS; and S-nitroso-N-acetylpenicillamine, a donor of NO [70–72]. Given that mammalian cells have RNA levels 4.4 times higher than that of DNA by weight, it could be simply presumed whenever DNA is damaged by such agents, RNA is surely damaged as well. However, ethyl methanesulfonate, a well-known DNA-damaging agent [73], lacked RNA damaging properties, while spermine and S-nitroso-N-acetylpenicillamine, although devoid of DNA damaging properties [74, 75], were able to damage RNA. Taken together, those data suggest that the formation of ROS and/or reactive nitrogen species may be critical for the reaction of a xenobiotic with RNA. Furthermore, they underline that the global impact of xenobiotics on nucleic acids cannot be defined by exploring exclusively the effects on DNA which, as it was shown, are not predictive of the RNA damaging potential of a chemical and* vice versa*.

From a pharmacological point of view, it is noteworthy that cancer cells are characterized by multiple DNA lesions resulting in transcription of large amounts of nonsense mRNA that is translated to proteins. Indeed, protein synthesis in cancer cells is stretched to its limit in contrast to normal cells [76]. The destruction of a significant portion of cellular RNA could lead to inhibiting the translation of critical proteins necessary for the proliferation and survival of cancer cells. On these bases, it is possible to postulate that the cytotoxic effect of RNA-damaging agents is selective for cancer cells.

RNA oxidation strategy can also help destroying viral RNAs by blocking the expression of virus-encoded essential proteins. An RNase P complexed with an external guide sequence was used with the aim of targeting the mRNA encoding the protease of human cytomegalovirus and thus blocking its expression and growth. The study reported a decrease of 95% in the protease expression and a decrease of 4 000 times in viral growth in cells infected with cytomegalovirus expressing the RNase P complexed with the external guide [77].

Moreover, it has recently been suggesting that host-encoded RNA molecules may play a role in the pathogenesis of the prion disease for the conversion of the normal prion glycoprotein, PrP^C^, to the aberrant prion protein, PrP^Sc^ [78]. This means that RNA degradation strategy can be useful in abrogating various RNA-associated diseases.

In conclusion, damage to RNA still represents a maturing approach and its true cellular potential remains to be deeply explored in both toxicological context and pharmacological context.
